# McCune-Albright Syndrome: A Case Series

**DOI:** 10.7759/cureus.101120

**Published:** 2026-01-08

**Authors:** Vishal Agarwal, Sambit Das, Amogh S Chappalagavi, Dayanidhi Meher, Devadarshini Sahoo

**Affiliations:** 1 Endocrinology, Diabetes and Metabolism, Kalinga Institute of Medical Sciences, Bhubaneswar, IND

**Keywords:** café-au-lait macules, fibrous dysplasia, mccune-albright syndrome, precocious puberty, recurrent fractures

## Abstract

McCune- Albright syndrome (MAS) is a rare disorder characterized by postzygotic somatic activation mutation of *GNAS, *leading to constitutively active Gsα and ligand-independent signaling of the Gs-coupled protein receptor. Polyostotic fibrous dysplasia, café-au-lait macules, and endocrine hyperfunction, commonly gonadotropin-independent precocious puberty (GIPP), are the three clinical hallmarks of this condition. Mosaicism results in a wide range of clinical presentations. In this case series, we present three cases of MAS with varied presentations. Case 1 describes a 9-year-old boy with recurrent fractures and craniofacial fibrous dysplasia. Case 2 is an 8-year-old girl with early menarche and GIPP. Case 3 details a 7-year-old girl with unilateral early breast development progressing from GIPP to gonadotropin-dependent precocious puberty (GDPP). As it is a heterogeneous disorder, it requires individualized management. Prompt recognition of clinical features can aid early diagnosis and intervention to mitigate disease progression.

## Introduction

McCune-Albright syndrome (MAS) is a rare, non-inherited, mosaic disorder resulting from postzygotic activating mutations in the *GNAS* gene, which encodes the alpha subunit of the stimulatory G protein (Gsα). This mutation leads to constitutive activation of adenylate cyclase and consequent elevation in cyclic adenosine monophosphate (cAMP) levels in affected tissues, driving autonomous cellular activity and the clinical manifestations of the disease [1]. First described in the 1930s, MAS is classically characterized by the triad of polyostotic fibrous dysplasia, café-au-lait macules, and endocrinopathies, most commonly gonadotropin-independent precocious puberty (GIPP). The true incidence of MAS is difficult to determine due to its mosaic nature and variable expressivity, but estimates suggest it occurs in approximately 1 in 100,000 to 1 in 1,000,000 live births. The syndrome affects individuals of all sexes and ethnicities equally and usually presents in early childhood, although clinical features may emerge over time depending on the distribution of the mutated cell lineages [2].

The clinical manifestations of MAS are diverse and often evolve with age. The skeletal involvement in the form of fibrous dysplasia may be monostotic or polyostotic, with a predilection for craniofacial bones, femur, tibia, ribs, and pelvis. Affected bones exhibit expansion, cortical thinning, and a characteristic ground-glass appearance on radiographs. These lesions may lead to bone pain, deformities such as shepherd’s crook deformity of the femur, pathologic fractures, and impaired mobility [3]. The cutaneous hallmark of MAS is the presence of café-au-lait macules with irregular, jagged borders, often described as having the “coast of Maine” appearance, which typically appear in infancy and respect the midline due to their mosaic pattern [4]. Endocrine manifestations are common and include autonomous ovarian estrogen production leading to precocious puberty in girls, as well as other endocrinopathies such as hyperthyroidism, growth hormone excess, neonatal Cushing syndrome, and phosphate-wasting disorders [5]. These abnormalities may coexist or evolve independently, further contributing to the phenotypic variability of MAS.

Here, we report three patients with MAS presenting with different clinical manifestations, highlighting its heterogeneous nature. Early diagnosis, regular surveillance, and individualized care are essential in improving the long-term outcomes of individuals with MAS. Further research is needed to develop targeted molecular therapies that can modify the course of this complex and heterogeneous disorder.

## Case presentation

Case 1

**Figure 1:**
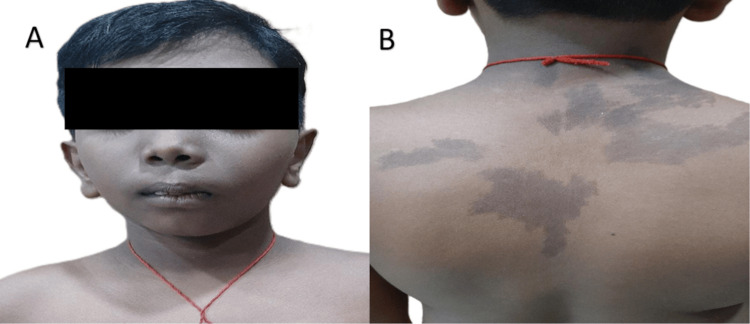
(A) Facial asymmetry; (B) multiple café-au-lait spots over the back.

A 9-year-old boy presented with complaints of pain in his left lower limb for the past 1 year. He had a history of multiple fractures, twice, in his left femur at the ages of 4 years and 5 years respectively, along with a history of fracture of the left humerus, which was operated on 1 year back. On examination, he had hyperpigmented macules with irregular margins over the posterior aspect of his trunk and right shoulder blade, along with asymmetric enlargement of the face (Figure 1).

His height was 133.4 cm (height Z-score −0.21), and his weight was 40.5 kg (weight Z-score + 1.31). There were no discriminatory features of Cushing syndrome, and there were no features suggestive of precocious puberty. Blood biochemistry showed normal serum total calcium and serum phosphate, with elevated alkaline phosphatase (Table 1). Hormonal workup revealed normal thyroid function test and normal plasma glucose profile (Table 1).

**Table 1:** Biochemical and hormonal parameters of case 1.

Parameter	Value	Reference Range
Serum total calcium (mmol/L)	2.4 (9.8 mg/dL)	2.07–2.60 (8.3–10.4 mg/dL)
Serum phosphate (mmol/L)	1.87 (5.8 mg/dL)	1.32–1.90 (4.1–5.9 mg/dL)
Alkaline phosphatase (U/L)	416	156–369
TSH (µIU/mL)	0.929	0.3–4.5
Free T4 (pmol/L)	14.9 (1.16 ng/dL)	10.3–25.7 (0.8–2.0 ng/dL)
Fasting plasma glucose (mmol/L)	4.9 (90 mg/dL)	<5.5 (<100 mg/dL)
Post-prandial plasma glucose (mmol/L)	5.7 (103 mg/dL)	<7.7 (<140 mg/dL)
Glycated hemoglobin (HbA1c, %)	5.3	<5.6

**Figure 2:**
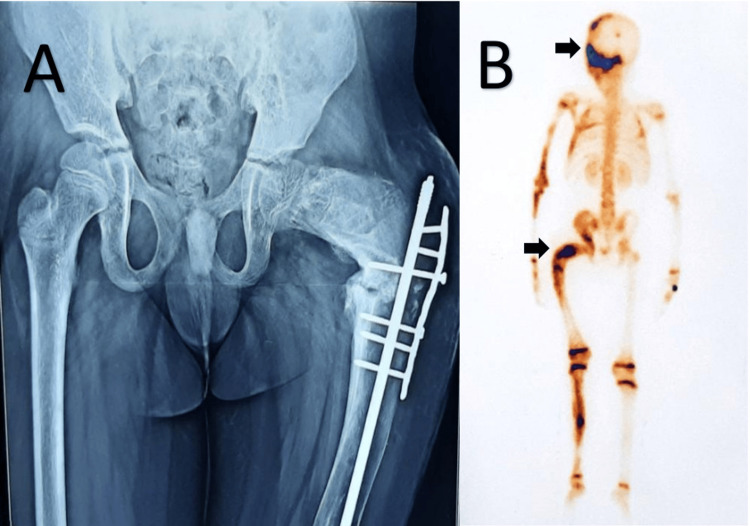
(A) Loss of trabecular pattern in left femur and typical ground-glass appearance with intramedullary nail in situ; (B) Bone scan (posterior view) showing increased osteoblastic activity in left hemicranium and femur (arrow).

X-ray pelvis (Figure 2a) showed loss of normal trabecular pattern in the femur with typical ground-glass appearance with nail plating (postoperative), and technetium-99 methylene diphosphonate (MDP) bone scan showed increased osteoblastic activity in left hemicranium, left orbit, maxilla, and femur (Figure 2b).

A diagnosis of MAS was made. The patient was started on zoledronic acid therapy (intravenously 5 mg) every 6 months, with symptomatic improvement 1 year post follow-up, with no recent episodes of fractures or bone pain and normal calcium profile.

Case 2

**Figure 3:**
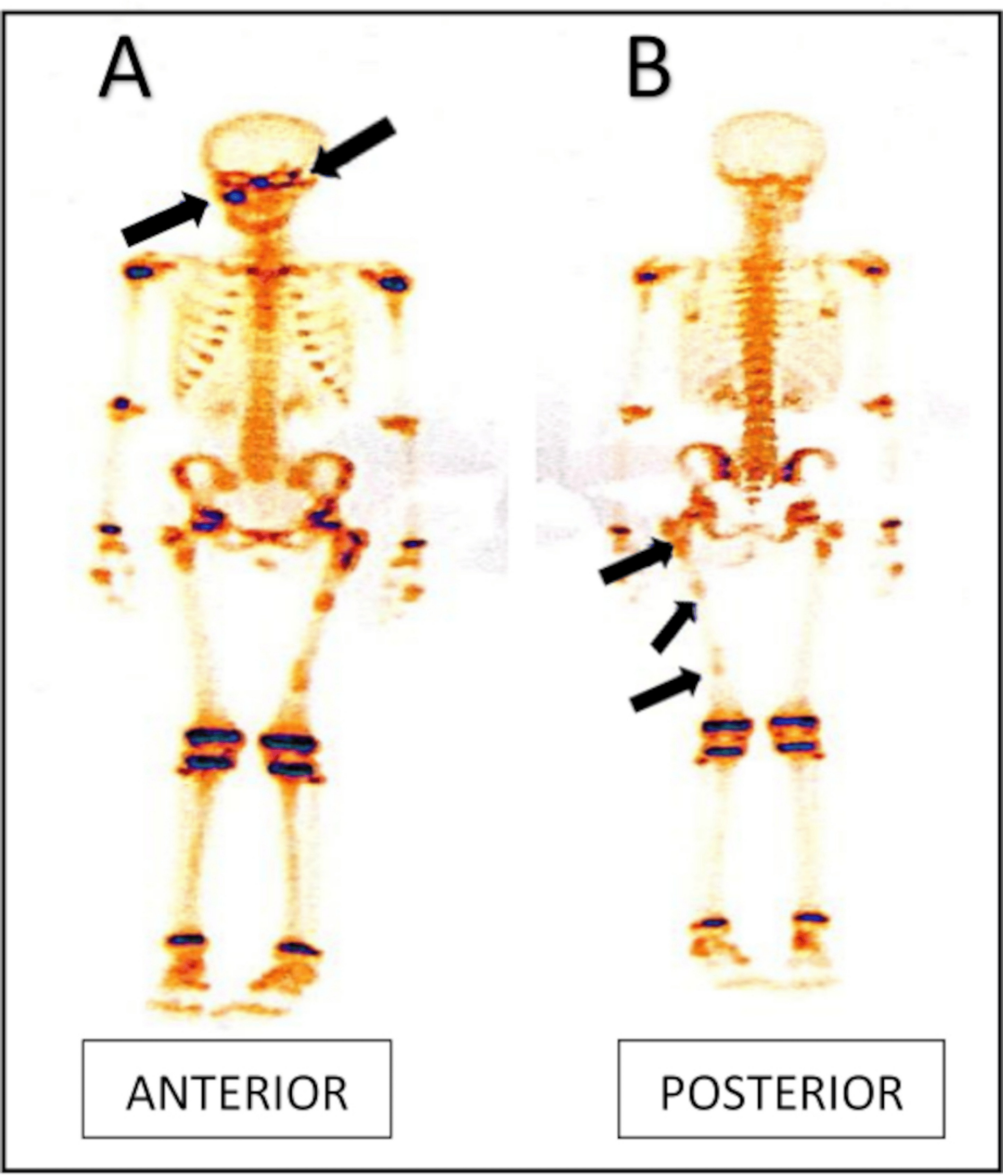
Bone scan showing multiple osteoblastic activity (arrow).

An 8-year-old girl presented with breast development for 5 years of age and vaginal bleeding for two days. On examination, she had an advanced height for age (144 cm; Z-score= +2.42), weight of 32.5 kg (Z-score= +1.1), and pubic hair of Tanner stage 2, along with Tanner stage 3 of breast development. No café-au-lait macules were noted. Her bone age was 13 years (assessed by the Greulich and Pyle method). On hormonal evaluation, her fasting baseline serum luteinizing hormone (LH) value was <0.1 mIU/mL (prepubertal), serum follicle-stimulating hormone (FSH) value was <0.1 mIU/mL (prepubertal), serum estradiol was 196 pg/mL (elevated; normal prepubertal: <10). A gonadotropin-releasing hormone (GnRH) stimulation test (20 µg/kg leuprolide IM) was done, which revealed a prepubertal LH and FSH response, suggestive of gonadotropin-independent peripheral precocious puberty (GIPP) (Table 2). On imaging, ultrasonography of pelvic structures showed uterine dimensions of 63 mm × 32 mm × 27 mm, a left ovarian simple cyst (28 mm × 30 mm), and the presence of an endometrial echo. Technitium-99m MDP bone scan revealed multiple areas of increased skeletal metabolism (Figure 3).

**Table 2:** Hormonal evaluation and imaging findings in case 2.

Parameter	Finding	Reference Range/Interpretation
Baseline hormonal evaluation		
Serum luteinizing hormone (LH)	<0.1 mIU/mL	Prepubertal
Serum follicle-stimulating hormone (FSH)	<0.1 mIU/mL	Prepubertal
Serum estradiol	196 pg/mL	Elevated (normal prepubertal: <10 pg/mL)
GnRH stimulation test (Leuprolide 20 µg/kg IM)		
Post-stimulation LH	0.11 mIU/mL	No pubertal response
Post-stimulation FSH	0.22 mIU/mL	No pubertal response
Pelvic ultrasonography		
Uterine size	63 × 32 × 27 mm	Enlarged for chronological age
Ovarian findings	Left ovarian simple cyst (28 × 30 mm)	Suggestive of autonomous estrogen production
Endometrium	Endometrial echo present	Estrogen effect
Skeletal imaging		
Tc-99m MDP bone scan	Multiple areas of increased skeletal metabolism	Consistent with polyostotic fibrous dysplasia

A diagnosis of MAS based on the presence of polyostotic fibrous dysplasia, as evidenced by a bone scan, along with the presence of GIPP. The patient was initiated on oral letrozole 2.5 mg once daily, oral medroxyprogesterone acetate 10 mg once daily, and alendronate therapy 35 mg once weekly and was planned for subsequent follow-up at 3-monthly intervals.

GIPP: gonadotropin-independent precocious puberty

Case 3

**Figure 4:**
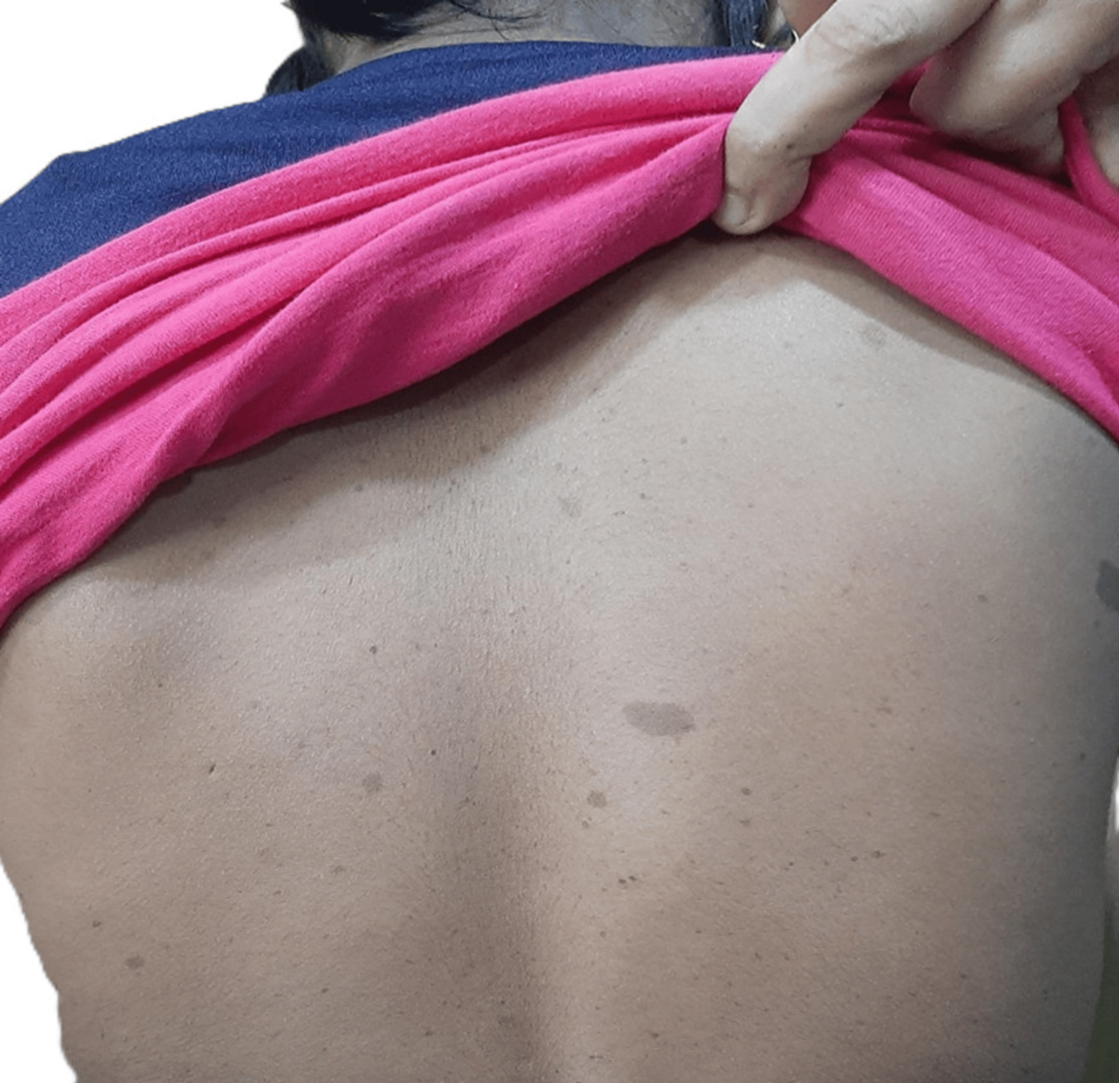
Multiple café-au-lait macules over the back.

A 7-year-old girl presented with unilateral early breast development. Café-au-lait macules were noted over the back (Figure 4).

Pubertal staging was Tanner stage 3 for breast and Tanner stage 1 for pubic hair. Her bone age was 12 years (assessed by the Greulich and Pyle method). Hormonal evaluation revealed serum LH and FSH of <0.1 mIU/mL (prepubertal), serum estradiol of 30 pg/mL (normal prepubertal: <10). A GnRH stimulation test was done, which revealed prepubertal LH and FSH, suggestive of GIPP (Table 3). The bone scan did not reveal any abnormal osteoblastic activity.

**Table 3:** Hormonal profile and imaging findings of case 3.

Parameter	Findings	Reference Range/Interpretation
Baseline hormonal evaluation		
Serum luteinizing hormone (LH)	<0.1 mIU/mL	Prepubertal
Serum follicle-stimulating hormone (FSH)	<0.1 mIU/mL	Prepubertal
Serum estradiol	30 pg/mL	Elevated (normal prepubertal: <10 pg/mL)
GnRH stimulation test(Leuprolide 20 µg/kg IM)		
Post-stimulation LH	0.23 mIU/mL	No pubertal response
Post-stimulation FSH	0.32 mIU/mL	No pubertal response
Skeletal imaging		
Tc-99m MDP bone scan	No abnormal osteoblastic activity	No evidence of fibrous dysplasia
Hormonal evaluation at 1-year follow-up		
Serum LH (fasting)	6.2 mIU/mL	Pubertal
Serum FSH (fasting)	12.5 mIU/mL	Pubertal
Serum estradiol	52 pg/mL	Pubertal range

A diagnosis of MAS based on the presence of café-au-lait macules and GIPP. In view of management, letrozole was initiated, but the patient was lost to follow-up. Upon return a year later, she had developed gonadotropin-dependent precocious puberty (GDPP) with fasting LH and FSH levels of 6.2 mIU/mL and 12.5 mIU/mL, respectively, along with serum estradiol of 52 pg/mL. She was started on GnRH agonist (injection leuprolide 3.75 mg IM once monthly) therapy in view of GDPP and planned for follow-up every 3 months.

Varied presentations of the three cases discussed above have been summarized in Table 4.

**Table 4:** Varied presentations of three cases

Clinical Presentation	Case 1	Case 2	Case 3
Cafe au lait macules	+	-	+
Polyostotic fibrous dysplasia	+	+	-
Precocious puberty	-	+	+
Other Endocrinopathies	-	-	-

## Discussion

Given the variable presentation, the differential diagnosis of MAS depends on the predominant clinical features. Conditions such as neurofibromatosis type 1 may be considered when café-au-lait spots are the presenting feature, although in NF1, the borders are smooth and “coast of California” in appearance and are often accompanied by axillary freckling and neurofibromas. Isolated precocious puberty may be seen in idiopathic forms or due to hypothalamic lesions. When skeletal lesions dominate the clinical picture, other entities such as monostotic fibrous dysplasia or other bone dysplasias should be considered. Disorders such as congenital adrenal hyperplasia or other mosaic syndromes like cutaneous skeletal hypophosphatemia syndrome may also mimic MAS in selected contexts [6,7].

Complications of MAS can be considerable, depending on the extent of systemic involvement. Recurrent fractures and progressive skeletal deformities can lead to chronic pain and disability, as seen in case 1. Endocrinopathies, if untreated, may result in metabolic consequences such as short stature, thyrotoxicosis, or cushingoid features. Craniofacial involvement can lead to vision or hearing loss. Rarely, malignant transformation of fibrous dysplasia to sarcoma has been reported, especially in patients exposed to radiotherapy [8,9]. Additionally, the psychosocial impact of the disease on quality of life, particularly in pediatric patients, warrants attention.

Diagnosis is established when at least two of the triad components (polyostotic fibrous dysplasia, café-au-lait macules, and endocrinopathies) are present. Fibrous dysplasia can affect any bone, leading to deformities, fractures, and functional impairment. Café-au-lait macules in MAS typically have irregular "coast of Maine" borders, distinguishing them from those in neurofibromatosis type 1 [10]. Fibrous dysplasia is relatively common and is characterized by fibrous tissue replacement of normal bone [11]. It can be an isolated condition or can present as a part of a syndrome such as MAS [12]. If only a single skeletal site is involved, the condition is known as monostotic fibrous dysplasia, whereas involvement of multiple sites leads to polyostotic fibrous dysplasia [13]. Skull base and proximal femur are the most commonly affected sites; however, any part of the skeleton can be involved, and thus the clinical presentation of fibrous dysplasia is highly variable and depends upon the location and extent of bone involved [14,15]. Endocrine abnormalities in MAS include GIPP, hyperthyroidism, Cushing’s syndrome, growth hormone excess, and hypophosphatemic rickets. GIPP, the most common endocrine manifestation, results from autonomous estrogen production in ovarian cysts or testicular Leydig cells, independent of gonadotropin stimulation [16]. Treatment strategies focus on symptom control. Bisphosphonates such as zoledronic acid are used for fibrous dysplasia to reduce fracture risk. Letrozole, an aromatase inhibitor, is preferred in GIPP to suppress estrogen production, while GnRH analogues like leuprolide are indicated in case the patient develops GDPP.

In the case of our series, a 9-year-old male child with fibrous dysplasia showed up with recurrent fractures and craniofacial involvement, along with multiple café-au-lait macules. His lack of endocrine problems supports the notion that skeletal involvement is usually the primary presentation of MAS. Polyostotic fibrous dysplasia is caused by abnormal Gs-alpha signaling in bone-forming cells, which causes fibro-osseous tissue to replace healthy bone. Affected people are more likely to experience frequent fractures, deformities, and functional impairment as a result. This patient's craniofacial fibrous dysplasia is especially worrisome since it may cause eyesight and hearing loss. Tc-99m MDP bone scan imaging, a crucial diagnostic tool for fibrous dysplasia, verified widespread involvement. Bisphosphonate therapy (zoledronic acid) was used as part of the management to lessen bone discomfort and turnover. While bisphosphonates are widely used, their long-term effectiveness in fracture prevention remains debated. In severe cases with progressive deformities, surgical intervention is warranted.

In the second case, an 8-year-old girl had premature thelarche, early-onset vaginal bleeding, and high estrogen levels. Her gonadotropin levels were reduced, which supported the diagnosis of GIPP, a defining endocrine abnormality characteristic of MAS. Autonomous ovarian cysts that release estrogen without gonadotropin stimulation are the source of GIPP in MAS. As a result, adult height gets compromised due to advanced bone age and premature epiphyseal fusion. This underscores the importance of early diagnosis and treatment in such patients. Bone scan results revealed that this patient also had polyostotic fibrous dysplasia, which is consistent with the traditional MAS triad. Aromatase inhibitors, such as letrozole, were used in management to lower estrogen synthesis and stop future pubertal development. The breakthrough bleeding was managed with medroxyprogesterone acetate. Aromatase inhibition continues to be the cornerstone of treatment because MAS-related GIPP is insensitive to GnRH analogues. Long-term monitoring for disease progression, including bone age assessment and endocrine function, is essential.

An unusual presentation, with initial unilateral breast enlargement along with café-au-lait macules but without polyostotic fibrous dysplasia, was demonstrated in the third case in our series. With decreased gonadotropins and increased estrogen levels, the preliminary hormonal assessment clinched the diagnosis of GIPP. However, on follow-up, increased levels of FSH and LH suggested that this patient transitioned to GDPP. This shift from GIPP to GDPP happens when the hypothalamic-pituitary-gonadal axis is prematurely activated by prolonged exposure to high estrogen levels. Leuprolide, a GnRH analog, is started in these situations to maximize eventual adult height and halt additional pubertal development. This case emphasizes how crucial longitudinal follow-up is for MAS patients who experience early puberty.

MAS is therefore a highly heterogeneous entity, with patients exhibiting varying degrees of skeletal, endocrine, and cutaneous involvement. Our case series demonstrates three different presentations: isolated polyostotic fibrous dysplasia, GIPP with polyostotic fibrous dysplasia, and evolving GIPP to GDPP with café-au-lait macules, highlighting the need for personalized management strategies.

## Conclusions

This case series highlights the heterogeneity of MAS presentations and underscores the importance of early diagnosis. Clinicians should maintain a high index of suspicion in patients with polyostotic fibrous dysplasias, café-au-lait macules, and endocrinopathies. A multidisciplinary approach is crucial for optimal management and to prevent complications.
